# Supplementary material for: HIVprotI: an integrated web based platform for prediction and design of HIV proteins inhibitors
Source: J Cheminform. 2018 Mar 9;10:12. doi: 10.1186/s13321-018-0266-y (PMC5845081; doi:10.1186/s13321-018-0266-y)
Supplement: Supplementary file 2 — Additional file 2. Source code of HIVProtI web server. [file 13321_2018_266_MOESM2_ESM.zip › HIVprotI_Source-code/hivprotI/weka-3-6-6/doc/weka/core/ClassloaderUtil.html]

ClassloaderUtil


---


|  |  |  |
| --- | --- | --- |
| **Overview** \| **Package** \| **Class** \| **Tree** \| **Deprecated** \| **Index** \| **Help** \| **Weka's home** | |  |
| **PREV CLASS**   **NEXT CLASS** | **FRAMES**    **NO FRAMES**     **All Classes** |  |
| SUMMARY: NESTED \| FIELD \| CONSTR \| METHOD | DETAIL: FIELD \| CONSTR \| METHOD |  |


---


## weka.core Class ClassloaderUtil

```
java.lang.Object
  weka.core.ClassloaderUtil
```

**All Implemented Interfaces:**: RevisionHandler

---

``` public class ClassloaderUtil extends java.lang.Object implements RevisionHandler ```

Utility class that can add jar files to the classpath dynamically.

**Version:**
:   $Revision: 5562 $

**Author:**
:   Mark Hall (mhall{[at]}pentaho{[dot]}org

---

**Constructor Summary**

`ClassloaderUtil()`


| **Method Summary** | |
| --- | --- |
| `static void` | `addFile(java.io.File f)`             Add file to CLASSPATH |
| `static void` | `addFile(java.lang.String s)`             Add file to CLASSPATH |
| `static void` | `addURL(java.net.URL u)`             Add URL to CLASSPATH |
| `java.lang.String` | `getRevision()`             Returns the revision string. |

**Methods inherited from class java.lang.Object**

`equals, getClass, hashCode, notify, notifyAll, toString, wait, wait, wait`

**Constructor Detail**

### ClassloaderUtil

```
public ClassloaderUtil()
```


**Method Detail**

### addFile

```
public static void addFile(java.lang.String s)
                    throws java.io.IOException
```

:   Add file to CLASSPATH

    :   **Parameters:**: `s` - File name **Throws:**: `java.io.IOException` - if something goes wrong when adding a file

---


### addFile

```
public static void addFile(java.io.File f)
                    throws java.io.IOException
```

:   Add file to CLASSPATH

    :   **Parameters:**: `f` - File object **Throws:**: `java.io.IOException` - if something goes wrong when adding a file

---


### addURL

```
public static void addURL(java.net.URL u)
                   throws java.io.IOException
```

:   Add URL to CLASSPATH

    :   **Parameters:**: `u` - URL **Throws:**: `java.io.IOException` - if something goes wrong when adding a url

---


### getRevision

```
public java.lang.String getRevision()
```

:   Returns the revision string.

    :   **Specified by:**: `getRevision` in interface `RevisionHandler`
    :   **Returns:**: the revision


---


|  |  |  |
| --- | --- | --- |
| **Overview** \| **Package** \| **Class** \| **Tree** \| **Deprecated** \| **Index** \| **Help** \| **Weka's home** | |  |
| **PREV CLASS**   **NEXT CLASS** | **FRAMES**    **NO FRAMES**     **All Classes** |  |
| SUMMARY: NESTED \| FIELD \| CONSTR \| METHOD | DETAIL: FIELD \| CONSTR \| METHOD |  |


---
